# Subtle changes in central dopaminergic tone underlie bradykinesia in essential tremor

**DOI:** 10.1016/j.nicl.2023.103526

**Published:** 2023-10-10

**Authors:** Donato Colella, Massimiliano Passaretti, Viviana Frantellizzi, Maria Silvia De Feo, Antonio Cannavacciuolo, Luca Angelini, Daniele Birreci, Davide Costa, Giulia Paparella, Andrea Guerra, Giuseppe De Vincentis, Alfredo Berardelli, Matteo Bologna

**Affiliations:** a Department of Human Neurosciences, Sapienza University of Rome, Italy; b Department of Clinical Neuroscience, Karolinska Institutet, Stockholm, Sweden; c Department of Radiological Sciences, Oncology and Anatomical Pathology, Sapienza University of Rome, Italy; d IRCCS Neuromed Pozzilli (IS), Italy; e Parkinson and Movement Disorder Unit, Study Center on Neurodegeneration (CESNE), Department of Neuroscience, University of Padua, Padua, Italy

**Keywords:** Bradykinesia, DAT-SPECT, Essential tremor, Parkinson’s disease

## Abstract

**Introduction:**

In this research, our primary objective was to explore the correlation between basal ganglia dopaminergic neurotransmission, assessed using 123I-FP-CIT (DAT-SPECT), and finger movements abnormalities in patients with essential tremor (ET) and Parkinson’s disease (PD).

**Methods:**

We enrolled 16 patients with ET, 17 with PD, and 18 healthy controls (HC). Each participant underwent comprehensive clinical evaluations, kinematic assessments of finger tapping. ET and PD patients underwent DAT-SPECT imaging. The DAT-SPECT scans were subjected to both visual and semi-quantitative analysis using DaTQUANT®. We then investigated the correlations between the clinical, kinematic, and DAT-SPECT data, in patients.

**Results:**

Our findings confirm that individuals with ET exhibited slower finger tapping than HC. Visual evaluation of radiotracer uptake in both striata demonstrated normal levels within the ET patient cohort, while PD patients displayed reduced uptake. However, there was notable heterogeneity in the quantification of uptake within the striata among ET patients. Additionally, we found a correlation between the amount of radiotracer uptake in the striatum and movement velocity during finger tapping in patients. Specifically, lower radioligand uptake corresponded to decreased movement velocity (ET: coef. = 0.53, p-adj = 0.03; PD: coef. = 0.59, p-adj = 0.01).

**Conclusion:**

The study's findings suggest a potential link between subtle changes in central dopaminergic tone and altered voluntary movement execution, in ET. These results provide further insights into the pathophysiology of ET. However, longitudinal studies are essential to determine whether the slight reduction in dopaminergic tone observed in ET patients represents a distinct subtype of the disease or could serve as a predictor for the clinical progression into PD.

## Highlights

• We correlated measurements of dopamine tone and finger tapping in ET and PD.
• We found heterogeneity in the radiotracer uptake within the striata in ET patients.
• Lower radioligand uptake corresponded to decreased movement velocity.
• The results provide further insights into the pathophysiology of ET.

## 1 Introduction

Essential tremor (ET) is a common movement disorder characterized by postural and kinetic tremor of the upper limbs (Bhatia et al., 2018). The presence of additional clinical symptoms, such as rest tremor or movement slowness (bradykinesia) (Bologna et al., 2023), in some cases presents challenges in differentiating it from Parkinson's disease (PD) (Algarni and Fasano, 2018, Tarakad and Jankovic, 2019). Various kinematic studies have demonstrated bradykinesia in ET patients (Costa et al., 2010, Jiménez-Jiménez et al., 2010, Goubault et al., 2017, Duval et al., 2006, Montgomery et al., 2000, Bologna et al., 2020, Angelini et al., 2023), as movement slowness without sequence effect (Bologna et al., 2020, Angelini et al., 2023, Paparella et al., 2023). While the role of the basal ganglia in the pathophysiology of bradykinesia in PD is widely recognized (Bologna et al., 2020, Berardelli et al., 2001), its possible involvement in ET patients is not as well-established (Paparella et al., 2021).

A potentially valuable instrumental method for studying the nigrostriatal dopaminergic system is 123I-Ioflupane dopamine transporter single-photon emission computed tomography (DAT-SPECT). Striatal dopamine transporter (DAT) radiotracer uptake indirectly assesses the extent of degeneration in dopaminergic terminals, indicative of PD and other parkinsonism (Benamer et al., 2000, Brogley, 2019). While DAT-SPECT scans are generally expected to yield negative results in ET patients, issues may arise where the definitive clinical diagnosis of ET remains uncertain even in the presence of a negative DAT-SPECT scan. (Menéndez-González et al., 2014). Also, when examining patients with isolated postural tremor, for example, DAT-SPECT revealed a slight reduction in radioligand uptake, albeit not statistically significant (Coria et al., 2012). Moreover, some studies have reported a significant decrease in dopamine transporter levels among patients with ET (Isaias et al., 2008, Schwartz et al., 2004). These findings may prompt questions about a shared pathophysiology between ET and PD, particularly in light of reported cases of ET patients progressing to PD (Thenganatt and Jankovic, 2016, Bologna et al., 2020, Bologna et al., 2020, Bologna et al., 2023, Paparella et al., 2021). However, uncertainties remain regarding whether individuals displaying subtle parkinsonian signs within the ET group represent potential prodromal cases of PD or constitute a distinct category that, in certain cases, could progress to PD.

Currently, the availability of an automated software, i.e., DaTQUANT (GE Healthcare) enables a more precise semi-quantitative assessment of dopaminergic terminal loss (Brogley, 2019, Neill et al., 2021). DaTQUANT results have confirmed the presence of intermediate 123I-Ioflupane uptake values in ET patients, which are between those observed in PD patients and healthy controls (HC) (Waln et al., 2015). However, it remains unclear whether this dopaminergic dysfunction correlates with the degree of movement slowness observed in ET. Although Schwartz et al. found a correlation between reduced ligand uptake values and impaired visuo-motor coordination (VMC) task performance in ET patients (Schwartz et al., 2004) it is worth noting that the VMC task is not specifically designed to assess bradykinesia, unlike the finger tapping movements, commonly employed in clinical settings (Bologna et al., 2020, Bologna et al., 2016).

The main objective of this study was to explore the potential association between changes in dopaminergic neurotransmission and the presence of bradykinesia in ET compared to PD and HC. To achieve this, we performed a kinematic analysis of repetitive finger tapping movements, allowing for an objective measurement of motor performance in ET patients. Furthermore, we quantitatively assessed the impairment of the dopaminergic system using DAT-SPECT imaging. Finally, we investigated potential correlations between kinematic parameters of finger movements and radioligand uptake values measured through DaTQUANT. Data obtained in patients with ET were compared to those of PD patients and normal subjects.

## 2 Materials and methods

### 2.1 Participants

For this study, 16 ET patients, 17 PD patients, and 18 HC were consecutively recruited from the Department of Human Neurosciences at Sapienza University of Rome. ET and PD diagnoses were performed using the current clinical criteria (Bhatia et al., 2018, Postuma et al., 2015). Notably, all PD patients enrolled in the study exhibited a tremor-dominant phenotype**.** We included ET patients, both with and without soft signs of parkinsonism, which encompasses pure ET and ET-plus patients (Bhatia et al., 2018). ET-plus patients presenting with additional soft signs, such as questionable dystonia, mild ataxia, or mild cognitive impairment, were excluded from the study. Patients with any other neurological or psychiatric conditions, as well as those with a history of taking anti-dopaminergic drugs, were also excluded. All patients underwent DAT-SPECT imaging as part of their diagnostic evaluation. We collected detailed demographic and clinical information for all participants. For ET patients, tremor-related therapy was discontinued at least 24 h before testing (Angelini et al., 2023, Paparella et al., 2023, Bologna et al., 2019, Bologna et al., 2018), (treatment details for ET patients are reported in Supplementary Table S1). PD patients were assessed in the practically defined OFF condition, i.e., after 12-hour withdrawal of dopaminergic therapy (Defer et al., 1999). The levodopa equivalent daily dose (LEDD) in PD is indicated in Table 1. Movement Disorder Society-sponsored revision of the Unified Parkinson's Disease Rating Scale, part III (MDS-UPDRS-III) (Antonini et al., 2013), to assess the severity of motor symptoms**,** as well as the Fahn-Tolosa-Marin Tremor Rating Scale (FTM-TRS) in patients with ET (Fahn et al., 1993). Clinical assessments included the evaluation of cognitive and psychiatric aspects and included the Montreal Cognitive Assessment (MoCA) (Freitas et al., 2013), and the Frontal Assessment Battery (FAB) (Dubois et al., 2000). HC were included based on the following criteria: a negative neurological examination and no history of taking anti-dopaminergic drugs. Written informed consent was obtained from all participants, and the study procedures were approved by the local ethics committee. The research was conducted following the principles outlined in the Declaration of Helsinki.

**Table 1:** Demographic, clinical, DaTQUANT data of patients with essential tremor (ET), Parkinson’s disease (PD) and healthy controls (HC).

	***ET***	***PD***	***HC***	***P***
**Age, years**	73.00 (68.50, 78.75)	73.00 (64.00, 78.00)	68.00 (65.00, 72.50)	0.222
**Sex, Female n, (%)**	7 (43.8)	5 (29.4)	5 (25.0)	0.469
**Disease duration, years**	9.00 (6.50, 12.00)“	3.00 (2.00, 6.00)		0.049
**Family history of tremor, n (%)**	**8 (50)**			
**FAB**	17.00 (16.75, 18.00)	16.00 (16.00, 17.00)*, #	17.00 (16.00, 18.00)	**0.009**
**MoCA**	26.00 (25.0, 26.0)	25.00 (23.00, 27.00)	27.5 (26.00, 28.3)	**0.029**
**MDS-UPDRS III**	9.50 (5.75, 17.50)	22.00 (16.00, 28.00)		**0.006**
***Bradykinesia subscores***	3.00 (0.0, 7.25)	9.00 (5.00, 13.00)		**0.006**
***Tremor subscores***	5.00 (2.75, 7.25)	7.00 (5.00, 9.00)		0.524
***Rigidity subscores***	0.00 (0.00, 1.00)	2.00 (0.00, 4.00)		**0.007**
**FTM-TRS**	19.5 (13.00, 27.00)			
**LEDD, mg/die**		242.5 (113–375)		
**Rest tremor**				
***Frequence, Hz***	5.79 (1.24)	5.67 (1.01)		0.998
***Magnitude,G-RMS^2***	0.36 (0.58)	0.45 (0.79)		0.738
**Postural tremor**				
***Frequence, Hz***	5.01(1.45)	5.15 (1.05)		0.680
***Magnitude,G-RMS^2***	1.72 (2.55)	0.48 (0.73)		0.042
**DaT-QUANT**				
***Striatum SBR***	3.04 (0.51)	1.86 (0.72)		**<0.001**
***Putamen SBR***	2.80 (0.52)	1.70 (0.75)		**<0.001**
***Caudate SBR***	3.28 (0.62)	2.18 (0.70)		**<0.001**
***Striatum z- score***	1.35 (1.02)	−0.87 (1.65)		**<0.001**
***Putamen z-score***	1.39 (0.88)	−0.79 (2.00)		**<0.001**
***Caudate z-score***	1.06 (1.30)	−0.55 (1.20)		**0.001**

The data are presented as mean (standard deviation) or median and interquartile range for non-parametric comparisons, if not otherwise specified. P-values results from groups comparisons, when group comparison resulted from Kruskal-Wallis analysis, post-hoc test Wilcoxon rank results were indicated if significant as: * for comparison with ET, **#** for comparison with HC FAB: Frontal Assessment Battery; FTM-TRS: Fahn-Tolosa-Marin Tremor Rating Scale; LEDD: Levodopa Equivalent Daily Dose; MDS-UPDRS III: Movement Disorder Society Unified Parkinson's Disease Rating Scale; MoCA: Montreal Cognitive Assessment; RMS: root-mean-square; SBR: striatal binding ratio. Bradykinesia subscores refer to the sum of the items from 3.4 to 3.8 and 3.14. Tremor subscores refer to the sum of the items from 3.15 to 3.18. Rigidity subscores refer to the item from 3.3.

## 3 Kinematic recordings and analysis

Bradykinesia was assessed through finger tapping movements. Participants sat comfortably on a chair and tapped their thumb on their index finger as fast as possible for 15 seconds, with a 60 second pause between movements to prevent fatigue (Bologna et al., 2020, Angelini et al., 2023, Paparella et al., 2023, Bologna et al., 2016, Colella et al., 2021, Paparella et al., 2021). Kinematic recordings were obtained using 3 infrared cameras (sampling frequency: 120 Hz) from the SMART motion system by BTS Technology, Italy. The cameras detected reflective markers placed on the participant’s thumb, index finger, and hand (acting as a reference plane). Considering our prior research, which consistently reported no significant influence of handedness on finger tapping execution, finger tapping assessment were conducted using the dominant hand for both ET patient and HC (Bologna et al., 2020, Paparella et al., 2023, Bologna et al., 2016). In PD patients, the markers were placed on the most affected side (Paparella et al., 2023, Bologna et al., 2016, Bologna et al., 2018). Offline movement analysis was performed using the SMART Analyzer software by BTS Engineering, Italy. Linear regression techniques were employed to determine the initial amplitude (degree) and velocity (degree/s) of the finger movement as well as the decrement in amplitude (degrees/number of movements) and velocity [(degrees/second) /number of movements] during repetitive movements. Again, the coefficient of variation (CV), calculated as the standard deviation divided by the mean value of the intertap intervals, was used to assess the motor rhythm. Higher CV values indicated less regular movement (Bologna et al., 2020, Bologna et al., 2016, Colella et al., 2021).

Tremor quantification in ET and PD patients was conducted by recording three 45-second epochs while patients were at rest or maintained anti-gravity posture with their arms extended forward (P1) flexed at the elbows (P2) (Bologna et al., 2020, Angelini et al., 2023, Bologna et al., 2019, Paparella et al., 2018) The root-mean-square (RMS) of the accelerometer traces in the three dimensions of space was measured for each reference marker positioned on the upper limbs to assess tremor amplitude. The amplitude was then expressed in G-RMS^2. The peak dominant frequency (Hz) of the postural tremor spectrum was also measured (Bologna et al., 2020, Bologna et al., 2019, Paparella et al., 2018, Bologna et al., 2015). Mean values between the two positions were considered for the subsequent analyses.

## 4 123I-FP-CIT (DAT-SPECT) acquisition and analysis

To minimize thyroid gland uptake, 400 mg of potassium perchlorate were administered to patients 30 min before intravenous injection of 185 MBq of 123I-FP-CIT Brain SPECT imaging was performed 3 h after the radiopharmaceutical injection using a dual-head gamma camera (Infinia, GE Healthcare, Milwaukee, USA) equipped with a low-energy parallel hole high-resolution (LEHR) collimator. The acquisition parameters included an angular step of 3 degrees, a 1.3 zoom factor, and a matrix size of 128 × 128. The energy window was set symmetrically to ± 10% of the 159-KeV 123I photopeak. SPECT images were acquired and reconstructed using the iterative ordered subset expectation maximization (OSEM) algorithm, and attenuation correction was applied using Chang's method. Initially, a qualitative visual assessment of the presynaptic nigrostriatal system was performed based on the DAT-SPECT images by the same operator. Then, a semiquantitative evaluation of the striatal distribution of 123I-FP-CIT was carried out using the specific DaTQUANT software (GE Healthcare). This software enabled accurate and fully automated registration of patients' SPECT images onto a 3D-volume model of interest derived from a large database of healthy individuals who had undergone brain DAT-SPECT scans and were matched by age to the patients. Specific binding ratio (SBR) values for each patient were obtained using the DaTQUANT software, calculated as the mean counts in the specific striatal region minus the mean counts in the background region divided by the mean counts in the background region. SBR values were determined for both the right and left striatal regions (Ferrazzano et al., 2020).

### 4.1 Statistical analysis

Gender differences between groups were assessed using Fisher's exact test. The differences in demographic and other clinical features among the groups were evaluated through the Kruskal-Wallis test and post-hoc pairwise comparisons using the Wilcoxon rank sum test. For the comparison of kinematic parameters between ET patients, PD patients, and HC, separate one-way ANOVAs were conducted with the factor 'GROUP'. Kinematic variables were assessed in separate ANOVAs. Post hoc analyses were performed using Tukey's honestly significant difference (HSD) correction for multiple comparisons. To analyse the differences in DaTQUANT data we applied *t*-test comparisons. Spearman’s correlation analysis was utilized to explore the relationship between clinical-demographic data and kinematic parameters or DaTQUANT parameters. Kinematic variables of interest were selected for finger tapping and tremor based on the results of group comparison analyses Pearson’s correlation was used to examine the relationship between movement kinematics and SBR values in patients’ contralateral putamen and caudate nuclei.

Unless otherwise stated, results were presented as mean values (standard deviation - SD) or median (interquartile interval) for clinical and demographic data. The significance level was set at p < 0.05. To control the overall Type I error rate resulting from multiple comparisons, we applied the false discovery rate (FDR) correction method (Curran-Everett, 2000). Data analysis was performed using STATISTICA® software (TIBCO Software Inc., Palo Alto, California, US) and implemented in RStudio (Posit team (2023), Integrated Development Environment for R. Posit Software, PBC, Boston, MA. URL https://www.posit.co/.).

## 5 Results

### 5.1 Clinical and demographic data

No significant differences were observed in terms of age and gender distribution between the three groups (Table 1). A family history of tremor was present in 8 out of 16 ET patients (50%), whereas none of the participants in our study had a family history of parkinsonism. Kruskal-Wallis for MoCA and FAB scores revealed significant group effect. Lower values were demonstrated for PD patients when compared to HC (p = 0.014) and ET (p = 0.014) for FAB scores, while MoCA post-hoc analysis did not display any significant result (Table 1). A significant difference (p = 0.02) was found in the MDS-UPDRS-III with PD patients exhibiting higher values than ET (Table 1). In ET patients, the MDS-UPDRS-III scores were mainly attributed to the presence of postural and kinetic tremor [median postural tremor scores in ET: 2.0 (1.0-2.0), in PD: 1.0 (1.0-2.0); median kinetic tremor scores in ET: 1.0 (0.0–1.0), in PD: 1.0 (0.0-1.75)]. Additionally, minimal subtle signs such as mild bradykinesia (6/16, 37.5%), incorrect posture (7/16, 47%), or minimal and uncertain rigidity of the upper limbs (5/16, 31.2%) contributed to the overall scores. On the other hand, the PD patients presented typical PD symptoms with rigidity, bradykinesia, and rest tremor, with a postural tremor in 13/17 patients (76.4%).

## 6 Kinematic analysis

The ANOVA showed a significant effect of the factor 'GROUP' on the number of movements (F_2,50_ = 13.38; P < 0.001), rhythm (F_2,50_ = 3.41; P = 0.041), amplitude (F_2,50_ = 3.59; P = 0.035) and velocity (F_2,50_ = 17.85; P < 0.001). Post-hoc analysis indicated that ET patients exhibited fewer movements compared to PD patients (P = 0.003) and HC (P < 0.001), while no difference was observed between PD patients and HC (P = 0.269) (Table 2). Velocity was significantly lower in the PD group (p < 0.001), and in ET compared to HC (p = 0.002). However, there was no significant difference in velocity between PD and ET patients (Table 2). The analyses for rhythm and amplitude showed no significant result. Comparison between PD and ET patients’ kinematic assessment demonstrated no differences in resting tremor but a greater postural tremor magnitude in ET patients (p = 0.046, Table 1).

**Table 2:** Kinematic variables of finger tapping in patients with essential tremor (ET), Parkinson’s disease (PD) and healthy controls (HC).

	***ET***	***PD***	***HC***	***F_(2, 50)_***	***P***	***P_1_***	***P_2_***	***P_3_***
**N° movements**	32.71 (8.91)	47.80 (13.25)	54.33 (14.45)	13.38	**<0.001**	**0.003**	**<0.001**	0.269
**Rhythm**	0.11 (0.06)	0.12 (0.05)	0.08 (0.03)	3.41	0.041	0.723	0.216	0.037
**Amplitude**	52.81 (10.14)	43.60 (11.96)	51.98 (11.14)	3.59	0.035	0.055	0.973	0.068
**Amplitude Slope**	−0.26 (0.27)	−0.16 (0.13)	−0.14 (0.17)	1.81	0.174	0.321	0.176	0.949
**Velocity**	897.60 (161.12)	774.30 (154.84)	1103.96 (188.16)	17.85	**<0.001**	0.104	**0.002**	**< 0.001**
**Velocity Slope**	-5.81 (4.30)	-3.47 (3.26)	-4.48 (4.45)	1.38	0.260	0.231	0.593	0.731

The rhythm is expressed as the coefficient of variation of the inter-tap intervals. The amplitude is expressed in degrees. The amplitude slope is expressed in degrees per number of movements. The velocity is expressed in degrees per second. The velocity slope is expressed in degrees per second per number of movements. Means and standard deviation are reported for each parameter for the three groups, with significant values in bold. F-values and P-values of ANOVAs, and P-values of the post-hoc Tukey's HSD test, P_1_ = comparison between ET and PD, P_2_ = comparison between ET and HC, P_3_ = comparison between ET and PD. Abbreviations: PD: Idiopathic Parkinson’s Disease, ET: Essential Tremor, HC: Healthy Controls.

## 7 DAT-SPECT

As expected, visual evaluation of DAT-SPECT scans showed asymmetric dopaminergic uptake in PD patients, resulting in positive findings for all cases, while none of the ET patients demonstrated qualitative reduction of DAT binding. Again, the semiquantitative DaTQUANT analysis demonstrated a statistically significant difference between the PD and ET groups for the striatum, putamen and caudate nuclei considering both SBR and z-scores values (Table 1).

## 8 Correlation analyses

**Fig. 1:**
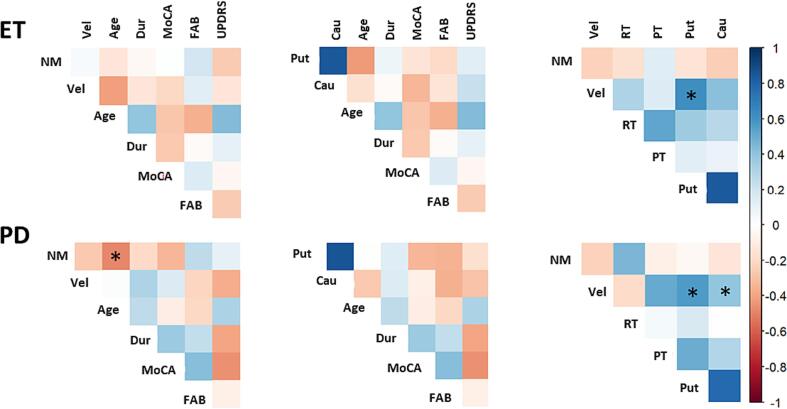
**Correlation matrices for Essential tremor (ET) and Parkinson’s disease (PD) patients.** The colour map indicates matrices of Pearson's correlations and coefficients, and significant p-values adjusted for false discovery rate (FDR) are indicated by asterixis. Cau: caudate striatal binding ratio; Dur: disease duration, FAB: Frontal Assessment Battery; MoCA: Montreal Cognitive Assessment; NM: finger tapping number of movements; PT: postural tremor root-mean-square; Put: putamen striatal binding ratio, RT: resting tremor root-mean-square, UPDRS: Movement Disorder Society Unified Parkinson's Disease Rating Scale; Vel: velocity of finger tapping.

Age correlated with the number of performed movements in PD patients (coef = -0.48, p-adj = 0.05) (Supplementary Table S2, Fig. 1). Conversely, no relationship was present between cognitive test scores or disease duration in ET and PD patients and finger tapping performance or tremor data (Supplementary Table S2-S3, Fig. 1). Also, there was no correlation between finger tapping velocity and postural and rest tremor magnitude, indicating that bradykinesia in ET and PD was not influenced by the presence of tremor (Supplementary Table S2-S3, Fig. 1). Pearson’s correlation analysis revealed a linear relationship between the velocity of finger tapping and the degree of DAT integrity, as indicated by the semi-quantitative analysis using DaTQUANT, in the contralateral basal ganglia. This correlation was observed for velocity in both PD and ET groups with the putamen SBR data (ET: coef = 0.53, p-adj = 0.03; PD: coef = 0.59, p-adj = 0.01;) and in the PD group for the caudate nucleus data (coef = 0.49, p-adj = 0.05) (Supplementary Table S2-S3, Fig. 1). However, no significant correlation was found between DaTQUANT data for the putamen and caudate nuclei, and MDS-UPDRS-III scores or the amplitude of postural and rest tremor in both ET and PD patients (Supplementary Table S2-S3, Fig. 1).

## 9 Discussion

Consistent with previous observations, the present study provides kinematic evidence of slowed finger tapping in ET patients compared to HC (Bologna et al., 2020, Angelini et al., 2023). As expected there was also kinematic evidence of reduced movement velocity during finger tapping in PD patients (Bologna et al., 2023). Again, the DAT-SPECT exams showed normal results in ET patients while altered findings in PD and the uptake values obtained through DaTQUANT analysis were consistently lower in the PD patient group compared to ET. Finally, we found a linear correlation between finger tapping movement velocity and the uptake values of the striatum, putamen and caudate nuclei as measured by the DaTQUANT demonstrated in both patients groups. Conversely, we did not find any correlation between DaTQUANT values and tremor in ET and in PD nor between finger tapping velocity and postural or kinetic tremor severity in patients, demonstrating that slowed movement velocity is not caused by the tremor itself (Goubault et al., 2017, Duval et al., 2006, Bologna et al., 2020).

The first novel finding of the present paper is the correlation between kinematic parameters in patients and the results of DAT-SPECT and DaTQUANT analysis. As expected, qualitative DAT-SPECT examinations revealed dysfunction of the nigro-striatal system in PD patients, whereas no visual abnormalities were observed in ET patients. This outcome was further supported by the semiquantitative DaTQUANT analysis, which demonstrated significantly lower overall uptake values in PD patients compared to the ET group (Brogley, 2019). Consistently with previous studies, ET patients evidenced normal DAT-SPECT scans based on visual interpretation (Kuya et al., 2018), while the DaTQUANT analysis revealed variations in DAT binding. These findings align with previous research indicating that reuptake values in patients with ET are lower than those in healthy individuals, but higher than in those with parkinsonian syndromes (Cuberas-Borrós et al., 2011, Isaias et al., 2010). The observed heterogeneity in DAT levels may reflect ET clinical and kinematic heterogeneity (Tarakad and Jankovic, 2019, Thenganatt and Jankovic, 2016), also confirming the pathophysiological link between ET and PD (Minen and Louis, 2008, Rajput et al., 2019, Pietracupa et al., 2021, Espay and Fasano, 2023). Notably, our findings revealed a significant correlation between velocity and the degree of dopaminergic reuptake in the caudate and putamen nuclei for both groups of patients. This correlation suggests that slower movement execution is associated with lower levels of dopaminergic reuptake in PD and ET. It has been well-established that nigrostriatal degeneration is the key factor contributing to bradykinesia in PD, and dopaminergic medications have shown significant efficacy in improving this symptom (Bologna et al., 2020). In contrast, the correlation between decreased movement velocity and dopaminergic dysfunction is less evident in ET, with the absence of clear clinical evidence of bradykinesia and the classical occurrence of qualitative negative DAT-SPECT scans. Although qualitative analysis of DAT-SPECT did not reveal dopamine dysfunction in our ET patients, the results of semiquantitative analyses and the correlations we found suggest a potential pathophysiological involvement of nigro-striatal circuits in some ET patients. However, this issue is still debated and requires further investigations (Erro et al., 2016).

Our results are consistent with a previous study by Schwartz et al., who demonstrated a reduction in putamen uptake in 8 out of 10 ET patients with impaired VMC, showing a linear correlation between DAT binding and contralateral performance (Schwartz et al., 2004). However, finger tapping, which represents the most used task for assessing bradykinesia in clinical evaluations, is more suitable for defining possible relationship with dopaminergic dysfunction in the basal ganglia, particularly the putamen, in ET patients. Notably, we have now observed a correlation between movement velocity and central dopaminergic tone in both groups of patients, further reinforcing the potential relationship between ET and PD. This finding may also possibly explain some ET patients progression to PD several years after the onset of symptoms (Thenganatt and Jankovic, 2016, Bologna et al., 2020, Bologna et al., 2020, Bologna et al., 2023, Paparella et al., 2021). However, it remains unclear whether ET patients with subtle parkinsonian signs are prodromal PD cases not fully manifested or if they represent a distinct entity that can, in some cases, evolve into PD. In this regard, earlier research has also indicated that ET may, to some extent, be a genetically-influenced condition, potentially falling under the category of repeat-expansion disorders, leading to a wide clinical overlap among various family members. This overlap includes some individuals affected by tremor-dominant PD and others by ET within the same family (Schneider et al., 2007). The discovery of patients with scans without evidence of dopaminergic deficit (SWEDD) adds complexity to this hypothesis. SWEDD can be observed not only in possible PD cases but also in those patients with no univocal parkinsonian signs (Schwingenschuh et al., 2010); including patients with adult-onset dystonic tremor resembling parkinsonian tremor (Hua and Lenz, 2005, Boecker et al., 2010). Moreover, new concepts suggest that complex interactions within motor networks may be more important than single structure impairment in causing motor symptoms, particularly bradykinesia (Bologna et al., 2023, Bologna et al., 2020). It has been hypothesized that in PD the cerebellum and cortical motor areas may play a compensatory role in movement feedback and compensation for impaired basal ganglia function during the execution of continuous and repetitive movements (Bologna et al., 2023, Bologna et al., 2020). However, it is still unknown whether an altered interplay between basal ganglia and cerebellum is causing altered voluntary movement execution in ET patients. Future functional magnetic resonance imaging studies will be necessary to confirm these hypotheses and better understand the diverse pathophysiological substrates underlying this heterogeneous clinical syndrome.

From a pathophysiological perspective, the results of our study could also be interpreted to gain insight into the mechanisms of action tremor in ET and PD. The cerebellum-thalamic-cortical circuit plays a key role in the pathophysiology of tremor in ET (Kosmowska and Wardas, 2021, Dirkx et al., 2018), while the contribution of the dopaminergic system in this condition is still unclear. Recent evidences suggest a dopamine modulation of cerebellar function through D3 receptors (Dirkx and Bologna, 2022). Rajput et al. found low dopamine levels in the caudate nucleus of ET patients (Rajput et al., 2019). Isaias and colleagues speculated on a connection between mild degeneration of the caudate nucleus's DAT and the onset of tremor in ET patients (Isaias et al., 2008, Isaias et al., 2010). Hence, dopaminergic dysfunction should contribute to the development of tremor in ET patients. However, we did not find any correlation between tracer uptake and the severity of action tremor in ET and PD patients. Moreover, we did not observe any correlation between DAT-SPECT values and the motor clinical severity, as measured by MDS-UPDRS-III, in PD (Curran-Everett, 2000, Hua and Lenz, 2005, Boecker et al., 2010) and ET patients (Bologna et al., 2020). In PD, pallidal dopamine depletion was demonstrated to facilitate rest tremor severity, but postural tremor appears to have mainly a nondopaminergic basis (Guerra et al., 2023, Berardelli et al., 2013). Therefore, further investigations are required to clarify whether effective dopaminergic alterations can influence cerebellar activity in the pathophysiology of action tremor in PD and ET.

Another innovative aspect of the study lies in the results obtained from the kinematic analysis of finger tapping, which provides valuable support to the clinical evaluation and diagnosis of patients by detecting specific movement parameters that are not easily recognized during a standard outpatient visit (Bologna et al., 2020, Bologna et al., 2020). In this regard, bradykinesia in PD patients is usually associated with hypokinesia (i.e., reduced movement amplitude) and, especially in the initial stages, with sequence effect, i.e., a progressive decrease in amplitude and speed during the execution of repetitive movements (Bologna et al., 2016, Paparella et al., 2021). However, our sample of PD patients did not show a sequence effect, probably due to the subtype enrolled in this study (Bologna et al., 2016). This study primarily included patients with PD who exhibited a tremor-dominant phenotype. During the initial phase, these patients presented diagnostic challenges, resembling ET, necessitating DAT-SPECT investigation. To the best of our knowledge, our study is the first to specifically investigate with objective techniques the bradykinesia features in patients with PD exhibiting a tremor-dominant phenotype. Overall, the results demonstrate that the sequence effect may lack in this subgroup of patients, as already described in patients with advanced PD (Bologna et al., 2023, Bologna et al., 2016).

Some possible confounding should be considered when interpreting the results of our study. Because we specifically tested finger tapping movements, we consider it unlikely that altered voluntary movement execution in ET is due to action tremor, which instead may possibly have a greater impact on proximal arm movements (Paparella et al., 2023, De Biase et al., 2022, Paparella et al., 2023, Bologna et al., 2020). As previously observed (Angelini et al., 2023, De Biase et al., 2022), in this study we found no correlation between action tremor severity and movement velocity during finger tapping in patients. Furthermore, concerning the ET group, we did not enroll patients with subtle dystonic symptoms, thus we can exclude that impaired motor performance was due, at least in part, to dystonia (Paparella et al., 2021, Lofredi et al., 2023). Concerning possible study limitations, the sample size was small, which may reduce the generalizability of our findings. Additionally, the assessment demonstrated a slight but significant cognitive reduction in PD patient (Schwingenschuh et al., 2010, Hua and Lenz, 2005). However, we excluded a significant confounding effect on motor assessment because this study did not include subjects with cognitive performance below the normal range. Furthermore, the correlation analysis did not show any impact of these tests on motor performance. Finally, DAT-SPECT was not acquired for HC, and this did not allow investigation of the relationship between movement kinematics and SBR values in the HC group. Further investigations should better delineate this issue, also considering that that mild parkinsonian syndromes may be present in up to 25% of elderly persons without PD (Postuma et al., 2015).

In conclusion, our study confirmed the presence of movement slowness in ET patients which correlates with subtle dopaminergic dysfunction. These results align with recent evidence indicating a significant overlap in the pathophysiology of some disorders of movement, including ET and PD [12,13,15,65]. We believe that the observation of shared phenomenological manifestations (lack of sequence effect of in PD patients with tremor-dominant phenotype as in ET) and partially overlapping pathophysiological mechanisms (role of central dopaminergic tone) that contribute to altered movement execution in both ET and PD supports the rationale for adopting the term 'bradykinesia' in reference to movement slowness in both conditions, as has been recently suggested (Bologna et al., 2023). Further studies, ideally incorporating kinematic techniques, are needed to understand better the role of dopaminergic dysfunction in the pathophysiology of bradykinesia in ET, PD, and other movement disorders.

## CRediT authorship contribution statement

**Donato Colella:** Conceptualization, Methodology, Investigation, Formal analysis, Writing – original draft. **Massimiliano Passaretti:** Conceptualization, Methodology, Investigation, Formal analysis, Writing – original draft. **Viviana Frantellizzi:** Methodology, Investigation, Formal analysis, Writing – review & editing. **Maria Silvia De Feo:** Methodology, Investigation, Formal analysis, Writing – review & editing. **Antonio Cannavacciuolo:** Investigation, Formal analysis. **Luca Angelini:** Investigation, Formal analysis. **Daniele Birreci:** Investigation, Formal analysis. **Davide Costa:** Investigation, Formal analysis. **Giulia Paparella:** Methodology, Investigation, Formal analysis, Writing – review & editing. **Andrea Guerra:** Formal analysis, Writing – review & editing. **Giuseppe De Vincentis:** Supervision, Writing – review & editing. **Alfredo Berardelli:** Supervision, Writing – review & editing. **Matteo Bologna:** Conceptualization, Methodology, Formal analysis, Visualization, Supervision, Writing – review & editing.

## Declaration of Competing Interest

The authors declare that they have no known competing financial interests or personal relationships that could have appeared to influence the work reported in this paper.

## Data Availability

Data will be made available on request.
