# Supplementary material for: Subtle changes in central dopaminergic tone underlie bradykinesia in essential tremor
Source: Neuroimage Clin. 2023 Oct 10;40:103526. doi: 10.1016/j.nicl.2023.103526 (PMC10587600; doi:10.1016/j.nicl.2023.103526)
Supplement: Supplementary data 1 [file mmc1.docx]

**Supplementary Table 1.** Pharmacological treatment in patients with essential tremor (ET).

| **ET** | **Treatment** |
| --- | --- |
| 1 | Propranolol 40mg |
| 2 | - |
| 3 | Bromazepam 1.5mg |
| 4 | - |
| 5 | - |
| 6 | - |
| 7 | Propranolol 40mg; Clonazepam 0.5mg |
| 8 | Propranolol 40mg |
| 9 | Clonazepam 0.3mg |
| 10 | Clonazepam 0.3mg |
| 11 | - |
| 12 | Propranolol 80mg |
| 13 | - |
| 14 | Propranolol 60mg |
| 15 | - |
| 16 | Propranolol 60mg; Clonazepam 3mg |

Data are expressed as daily dose.
