# Supplementary material for: Subtle changes in central dopaminergic tone underlie bradykinesia in essential tremor
Source: Neuroimage Clin. 2023 Oct 10;40:103526. doi: 10.1016/j.nicl.2023.103526 (PMC10587600; doi:10.1016/j.nicl.2023.103526)
Supplement: Supplementary data 2 [file mmc2.docx]

**Supplementary Table 2** Correlations of clinical, kinematic, and DaTQUANT data in Parkinson’s disease patients.

|  | **Age** | **Duration** | **MOCA** | **FAB** | **MDS-UPDRS III** | **Rest tremor RMS** | **Postural tremor (P2) RMS** | **Putamen SBR** | **Caudate SBR** |
| --- | --- | --- | --- | --- | --- | --- | --- | --- | --- |
| **Velocity** |  |  |  |  |  |  |  |  |  |
| *Coef* | 0.00 | 0.36 | 0.15 | 0.04 | -0.39 | -0.18 | 0.51 | 0.59 | 0.49 |
| *P-adj* | 1.00 | 0.16 | 0.58 | 0.87 | 0.12 | 0.57 | 0.07 | **0.01** | **0.05** |
| **N° Movemements** |  |  |  |  |  |  |  |  |  |
| *Coef* | -0.45 | -0.30 | -0.16 | 0.32 | 0.15 | 0.45 | -0.14 | -0.06 | -0.13 |
| *P-adj* | **0.07** | 0.25 | 0.54 | 0.21 | 0.56 | 0.14 | 0.64 | 0.81 | 0.62 |
| **Putamen SBR** |  |  |  |  |  |  |  |  |  |
| *Coef* | 0.05 | 0.19 | -0.31 | -0.25 | -0.16 | 0.16 | 0.43 | - | - |
| *P-adj* | 0.84 | 0.48 | 0.22 | 0.34 | 0.54 | 0.62 | 0.13 | - | - |
| **Caudate SBR** |  |  |  |  |  |  |  |  |  |
| *Coef* | -0.11 | 0.28 | -0.14 | -0.30 | -0.19 | -0.01 | 0.28 | - | - |
| *P-adj* | 0.67 | 0.27 | 0.60 | 0.24 | 0.46 | 0.98 | 0.33 | - | - |

Results of Pearson’s correlations are presented as coefficient and p-adjusted for false discovery rate (FDR). FAB: Frontal Assessment Battery; MOCA: Montreal Cognitive Assessment; MDS-UPDRS III: Movement Disorder Society Unified Parkinson's Disease Rating Scale; P2: posture 2, RMS: root-mean-square; SBR: striatal binding ratio.
