# Supplementary material for: Subtle changes in central dopaminergic tone underlie bradykinesia in essential tremor
Source: Neuroimage Clin. 2023 Oct 10;40:103526. doi: 10.1016/j.nicl.2023.103526 (PMC10587600; doi:10.1016/j.nicl.2023.103526)
Supplement: Supplementary data 3 [file mmc3.docx]

**Supplementary Table 3** Correlations of clinical, kinematic, and DaTQUANT data in Essential Tremor patients.

|  | **Age** | **Duration** | **MOCA** | **FAB** | **MDS-UPDRS III** | **Rest tremor RMS** | **Postural tremor (P2) RMS** | **Putamen SBR** | **Caudate SBR** |
| --- | --- | --- | --- | --- | --- | --- | --- | --- | --- |
| **Velocity** |  |  |  |  |  |  |  |  |  |
| *Coef* | -0.29 | -0.06 | -0.05 | 0.14 | -0.17 | 0.20 | 0.14 | 0.53 | 0.35 |
| *P-adj* | 0.28 | 0.84 | 0.85 | 0.72 | 0.53 | 0.46 | 0.63 | **0.03** | 0.18 |
| **N° Movemements** |  |  |  |  |  |  |  |  |  |
| *Coef* | -0.19 | -0.09 | -0.05 | 0.14 | 0.01 | -0.21 | 0.14 | -0.25 | -0.32 |
| *P-adj* | 0.49 | 0.74 | 0.86 | 0.72 | 0.96 | 0.43 | 0.64 | 0.36 | 0.23 |
| **Putamen SBR** |  |  |  |  |  |  |  |  |  |
| *Coef* | -0.25 | 0.12 | -0.13 | 0.02 | 0.14 | 0.34 | 0.12 | - | - |
| *P-adj* | 0.66 | 0.34 | 0.95 | 0.62 | 0.61 | 0.20 | 0.68 | - | - |
| **Caudate SBR** |  |  |  |  |  |  |  |  |  |
| *Coef* | -0.16 | 0.14 | -0.38 | 0.04 | 0.25 | 0.29 | 0.08 | - | - |
| *P-adj* | 0.63 | 0.57 | 0.98 | 0.15 | 0.35 | 0.27 | 0.78 | - | - |

Results of Pearson’s correlations are presented as coefficient and p-adjusted for false discovery rate (FDR). FAB: Frontal Assessment Battery; MOCA: Montreal Cognitive Assessment; MDS-UPDRS III: Movement Disorder Society Unified Parkinson's Disease Rating Scale; P2: posture 2, RMS: root-mean-square; SBR: striatal binding ratio.
